# Patient perspectives of nuisance bleeding and adherence to dual antiplatelet therapy: a qualitative study

**DOI:** 10.1136/openhrt-2020-001405

**Published:** 2020-10-15

**Authors:** Christalla Pithara, Maria Pufulete, Thomas W. Johnson, Sabi Redwood

**Affiliations:** 1 National Institute for Health Research Applied Research Collaboration West (NIHR ARC West) at University Hospitals Bristol and Weston NHS Foundation Trust, Bristol, UK; 2 Population Health Sciences, University of Bristol, Bristol, UK; 3 Bristol Trials Centre (Clinical Trials and Evaluation Unit), University of Bristol, Bristol, UK; 4 Department of Cardiology, Bristol Heart Institute, Bristol, UK

**Keywords:** antiplatelet treatment, quality of care and outcomes, education, delivery of care

## Abstract

**Objective:**

To understand the experiences of patients with dual antiplatelet therapy (DAPT) and nuisance bleeding, and their perspectives of the impact of nuisance bleeding on medication adherence and information seeking.

**Methods:**

We conducted focus groups with patients who had undergone percutaneous coronary intervention, coronary artery bypass graft and conservatively managed acute coronary syndrome patients. Two focus groups were with patients at the early stages of treatment (0–3 months), and two with patients coming to the end of treatment (9–12 months). Group discussions were audio recorded, and recordings were transcribed verbatim, anonymised and analysed using framework analysis.

**Findings:**

Nine patients taking DAPT for up to 3 months, and 12 taking DAPT between 9 and 12 months participated in the focus groups. We found that: (1) participants adhered to treatment when they believed DAPT was important to health outcomes; (2) those who experienced nuisance bleeding reported symptoms to be mild and manageable; (3) participants’ and their family’s understanding of DAPT risks and benefits, and their ability to manage symptoms, influenced perspectives of and experiences with adherence. Factors influencing DAPT knowledge included access to medication counselling, engaging with information communicated during medication counselling, and access to timely, relevant and expert information and advice after discharge from hospital.

**Conclusions:**

Positive attitudes towards adherence were facilitated by knowledge and understanding of DAPT and confidence in dealing with symptoms caused by DAPT, but hindered by lack of opportunities to access relevant, timely and appropriate medication counselling. Education interventions should aim to support medication literacy through family-centred approaches and involve patients and families at all stages of intervention design and evaluation.

**Key questions**

**What is already known about this subject?**

- Following percutaneous coronary intervention, coronary artery bypass grafting and medical management of acute coronary syndrome, patients need to adhere to dual antiplatelet therapy (DAPT), the recommended treatment for prevention of secondary ischaemic events. DAPT is associated with bleeding, which might result in discontinuation and non-adherence. So far studies have tended to focus on bleeding that requires medical attention, but it is not well understood how and whether nuisance bleeding impacts on adherence and access to information.

**What does this study add?**

- This study highlights the value of patient-centred medication counselling, that is available throughout patients’ care and treatment journey. Participants were willing to accept nuisance bleeding when they understood this to be a side effect of DAPT, and believed DAPT was important for their recovery. There were barriers, however, to accessing relevant, timely and expert information and advice in both hospital and community settings. Comorbidities and the need to manage complex medication regimens influenced adherence and symptom recognition. Family support was an important facilitator to adherence.

**How might this impact on clinical practice?**

- Medication counselling needs to address patient and family medication literacy needs and should be provided in a time-appropriate and needs-appropriate way. Patients should have opportunities to access medication information and counselling for the duration of their treatment, transitioning between primary and secondary care settings.

## Introduction

Patients’ ability to self-manage coronary heart disease (CHD) is crucial for patient independence, secondary prevention and maintaining quality of life.^1^ Self-management refers to ‘decision-making and behaviours performed by individuals to manage illness on a daily basis, and promote health’,^2^ and includes medication adherence and help-seeking behaviours, for example, access to information and care. Following coronary interventions, for example, percutaneous coronary intervention (PCI), coronary artery bypass grafting (CABG) and medical management of acute coronary syndrome (ACS), patients need to adhere to dual antiplatelet therapy (DAPT), the recommended treatment for prevention of secondary ischaemic events.^3 4^ DAPT is a combination of aspirin and a second antiplatelet agent (clopidogrel, ticagrelor or prasugrel), and is taken, in some cases alongside an anticoagulant, for up to a year.^3 4^

DAPT is associated with improved CHD outcomes^3^ but exposes patients to an elevated risk of bleeding, especially if combined with an anticoagulant.^5^ Bleeding has been linked to non-adherence and worsening quality of life.^6^ Non-adherence to secondary prevention medication such as DAPT is common,^7 8^ with one study reporting DAPT non-adherence in about 48% of patients.^7^ Even though more severe forms of bleeding are well reported in randomised controlled trials, impact of nuisance bleeding, described as non-actionable bleeding^9^ that includes nosebleeds, bleeding from gums and excessive bruising, on real-life patient behaviours is not as well understood.^6 10^

To our knowledge, only one study has specifically explored patient experiences of nuisance bleeding and perspectives on adherence when on DAPT.^11^ Understanding patient experiences with treatment and side effects can help identify mediating factors related to sustaining adherence and help improve patient education and empowerment.^1^ Studies evaluating patient education interventions have reported little evidence of effectiveness in improving adherence rates.^2 12 13^ One criticism has been that interventions have been professional, rather than patient-led, meaning that patients’ needs and preferences are not always addressed.^2 14^

We report on findings from a qualitative study exploring patients’ perspectives of DAPT and nuisance bleeding, and of factors influencing responses to nuisance bleeding, focusing on adherence and help-seeking behaviours. Qualitative methods are ideal for exploring patient perspectives, beliefs and motivations shaping individual behaviour, including health and illness behaviours.^15^ This qualitative study formed part of the ADAPTT study,^6 16^ designed to investigate incidence of bleeding in a real world population with and without ACS exposed to different DAPT regimens. This qualitative study was designed following discussions with a patient and public involvement (PPI) group whose role was to provide ongoing feedback and advice to the ADAPTT research team on study design and findings,^6 16^ after the group flagged up their own experiences of nuisance bleeding symptoms.

## Methods

### Patient and public involvement

A patient advisory group (consisting of PCI, CABG and conservatively managed patients with ACS, which formed the three study cohorts of the ADAPTT study) provided input throughout this qualitative study. The group attended regular meetings to discuss results from the study and provide feedback to findings.^6^

### Setting

This study was undertaken in the West of England, UK. Patients approached for recruitment had received care from one cardiology department in the West of England. Patient data collection took place on University of Bristol premises.

### Study design and sample

Four focus groups were assembled, including patients (a mixture of post-CABG surgery, post-PCI and medically treated ACS) at the early stages of DAPT treatment (0–3 months into treatment), and those at the end of DAPT treatment (taken DAPT for 9–12 months). Focus groups were used to identify the range of views and experiences of patients^17^ with regard to: attribution of symptoms to DAPT; range of thresholds for seeking further information and help; range of thresholds for requesting a change in medication; and issues related to adherence and quality of life.

### Patient recruitment

A detailed description of the patient recruitment process is reported elsewhere.^16^ Patients were approached by research nurses and consultant cardiologists during follow-up and postsurgery clinics, cardiac rehabilitation sessions and day clinics. Those who expressed interest received an invitation letter and patient information leaflet explaining the study in more detail and provided researcher contact details, and focus group dates. Approximately 1 week after initial contact, potential focus group participants were contacted again by members of the ADAPTT study team to confirm attendance. On the day of the focus groups two qualitative researchers, members of the National Institute for Health Research Applied Research Collaboration West (NIHR ARC West) answered questions and addressed concerns specific to the study, data collection process and use of data, before participants formally consented to take part by completing an informed consent form.

### Data collection

Focus groups were conducted between June and July 2017. Three experienced qualitative researchers cofacilitated the four focus groups (two cofacilitators were present at each focus group; CP was present during all focus groups). A topic guide (online supplemental material) was used to explore the following:

10.1136/openhrt-2020-001405.supp1 Supplementary data

1. Participants’ views on and satisfaction with the information they were given on DAPT; information seeking and perceived information needs.
2. Issues related to adherence.
3. Issues related to bleeding and accessing healthcare professionals to discuss medication.

Each focus group lasted between 1 and 1.5 hours. All focus groups were recorded using an encrypted digital audio recorder, and audio files were fully transcribed, anonymised and checked for accuracy.

### Qualitative data analysis

Anonymised focus group transcripts were imported into QSR NVivo V.11 data management software to aid data coding and management, and analysed using a framework approach.^18^ Focus group data were initially coded deductively, guided by the topics informing group discussions. In iterative rounds of analysis, further codes were inductively created within these initial categories to reflect the issues spontaneously raised by participants during the discussion, which were subsequently categorised under higher order themes. Using a framework approach,^18^ data were scrutinised for differences and similarities within and across themes, coronary intervention group (PCI, CABG and ACS) and focus groups/ time frames for antiplatelet therapy. One researcher (CP) led the analysis, with the coding frame being developed in collaboration with the coinvestigators. The team met regularly to discuss the coding framework and themes, and any implications for ongoing data collection, to ensure trustworthiness, credibility and rigour.

## Results

### Participants

Twenty-one patients were recruited, the majority of which had received PCI and CABG interventions, and only one was medically treated ACS (table 1 for patient demographic characteristics). Nine participants had been taking DAPT for up to 3 months, and 12 between nine and 12 months. Of the 21 participants, only one was a woman. Average age was 66 years, ranging between 48 and 88 years of age. Participants recruited represented the spectrum of social deprivation categories captured by the Index of Multiple Deprivation, a relative measure of social deprivation of neighbourhoods in the UK, with equal numbers of participants recruited from the most and least deprived areas. The spouse of one participant attending one of the two ‘0–3 months’ groups chose to sit in during the focus group and contributed to the discussion but is not included in table 1. Four out of nine participants in the ‘0–3 months’ group, and 6 out of 12 participants in the ‘9–12 months’ group reported experiencing ‘nuisance bleeding’. Three participants in the ‘0–3 months’ group and two in the ‘9–12 months’ group reported that they had not experienced any side effects from their DAPT medication.

**Table 1:** Participant demographic information

0–3 months into DAPT	N=9
Average age: 65 years (48–84)	
Male	8/9
Ethnicity	
White British	9
PCI	5/9
CABG	4/9
IMD Quintiles	
1 (least deprived)	1/9
2	2/9
3	3/9
4	2/9
5 (most deprived):	1/9
9–12 months into DAPT	N=12
Average age: 67 years (50–88)	
Male	12
Ethnicity	
White British	12
PCI	9/12
CABG	2/12
ACS	1/12
IMD quintiles	
1 (least deprived)	3/12
2	2/12
3	2/12
4	2/12
5 (most deprived)	3/12

ACS, acute coronary syndrome; CABG, coronary artery bypass graft; DAPT, dual antiplatelet therapy; IMD, Index of Multiple Deprivation; PCI, percutaneous coronary intervention.

### Findings

Comparisons between the narratives of patients from the three coronary intervention groups, and those at either of the two stages of treatment, did not highlight any differences in attitudes or beliefs about adherence and information seeking. Most participants from the two treatment duration groups reported nuisance bleeding to be mild and not result in non-adherence. Differences in experiences and perspectives between groups are reported where relevant

#### Knowledge and understanding

Medication information was mostly communicated before patients were discharged from hospital, and most were aware of the bleeding risk associated with DAPT. Most would attribute symptoms to antiplatelet medication: ‘I would put (nuisance bleeding) down to the (anti)platelets straight away.’ (102_Male_73yrs; PCI; 0 to 3 months into treatment). Understanding the cause of nuisance bleeding could be reassuring.

When I was given the medication at the hospital […] the nurse did explain what it was and what would happen, so it wasn’t a surprise that I was bleeding more. I understand what was causing it so that was fine (106_Male_55yrs; PCI; 0 to 3 months into treatment).

It could also inform adherence-specific decision-making when experiencing nuisance bleeding. Participants considered disease severity and health implications from discontinuing treatment, against the severity of nuisance bleeding.

I had problems initially coming to terms with the fact that I actually needed a stent, but when I had come to terms with that, taking antiplatelet therapy was not a problem, the implications of not taking it are too severe to consider anything else (205_Male_72yrs; PCI; 9 to 12 months into treatment)

Involvement of family members in adherence was also discussed. Family members in some cases took control of the patient’s care by preparing and dispensing medication, and encouraging patients to adhere to treatment.

Our daughter […] said to me, ‘Mum, make sure dad does this.’ And she’s done a list and put them all in these little boxes (wife of 101; PCI; 0 to 3 months into treatment)

Patients with a family history of CHD recounted family stories to emphasise potential consequences of medication non-adherence.

My sister, […], had never had any heart conditions but had been on statins because […] of our family history and she stopped taking the statins […]she ended up having a triple by-pass […] (statins) would probably have prevented that (105_Male_58yrs; PCI; 0 to 3 months into treatment)

Most participants who had experienced nuisance bleeding were not concerned about their symptoms, and shared strategies used to minimise the impact on quality of life.

I’m not shaving very much and gone back to the electric (razor) because when you do cut yourself, obviously you bleed a little bit, […] but […] no (bleeding is not a concern) (106_Male_55yrs; PCI; 0 to 3 months into treatment)

The short-term duration of DAPT, meaning that symptoms would only last for the duration of treatment, was another catalyst. ‘I am certainly not stopping the Ticagrelor because I am only going to get it for 12 months’ (206_Male_70yrs; PCI; 9 to 12 months into treatment)

(Prior to treatment) I had slight problems with haemorrhoids. […] With taking both medications, the aspirin and the other, […] it did increase the bleeding there. I was quite concerned about it. But now that I’ve been off the tablet […] it’s all improved. (220_Male_88yrs; PCI; 9 to 12 months into the treatment)

Some shared hypothetical scenarios to illustrate when nuisance bleeding would raise concerns and trigger access to care.

With my gums and that, if it bled and bled and bled for two or three days then I probably would phone a doctor (202_Male_64yrs; CABG; 9–12 months into treatment)

If you were bleeding, I would think […] minor things, but not if you can’t stop. If it was more serious, I think I would be (calling emergency services) (204_Male_61yrs; PCI; 9 to 12 months into treatment)

Some participants compared DAPT to other medication such as statins and beta-blockers. Such agents were reported to have more debilitating side effects, or thought to be less important to recovery: ‘At the moment, I feel (DAPT) is a more important drug’ (206_Male_70yrs; PCI; 9 to 12 months into treatment)

Statins […] causes me to sleep too much […] but if they do cause issues like that, particularly like sleeping, then I stop, but with the blood ones, […] (nuisance bleeding) wasn’t a consideration to stop. (204_Male_61yrs; PCI; 9 to 12 months into treatment)

#### Accessing medication information

##### Experiences with medication counselling in hospital settings

Participants’ experiences with medication counselling differed even with participants being counselled in the same setting. Not all felt their information needs were addressed during these encounters.

Focus Group 01_patients 9-12 months into their treatment

204_Male_61yrs; PCI: I can’t remember my consultant telling me any of the side effects that may well happen to be quite honest with you […]

206_Male_70yrs; PCI: (I was given) loads of information. Explanations on each pill and an indication of how long they should be taken, certainly in my case.

207_Male_71yrs; PCI: The only time I basically knew how long I had to take mine for was because it was on the pillbox, take until 5 June

The only female participant believed this information was directed towards male patients, whereas female-specific needs were poorly addressed.

The information in the leaflet […] was directed at men […] from a woman’s point of view there needs to be a lot more information for us (109_Female_54yrs; CABG; 0–3 months into treatment)

The timing of medication counselling was another factor impacting on knowledge levels. Several participants recounted being approached during their stay in hospital at a time when they physically and emotionally felt unable to engage in medication counselling with health professionals.

[The surgeon] came round after my surgery but I was completely out of it. […] he said ‘you’re ok, everything was successful […] Do you have any questions? Well I had so much morphine. […] I couldn’t focus mentally. (109_Female_54yrs; CABG; 0–3 months into treatment)

Focus group 03_ patients 9-12 months into treatment

215_Male_68yrs; PCI: The trouble is when you’re given the medication you’re ill. […] You’ve just had a bloody heart attack.

216_Male_78yrs; ACS: And you’ve got a lot more things on your mind than worrying about (the side effects of the medication).

##### Accessing information in community settings

A minority of participants were referred to cardiac rehabilitation clinics after CABG. Interactions with peers and health professionals in this setting were an important source of treatment and side-effect information.

When somebody was talking in my rehab group they’d brought up the thing of bruising and I thought ‘oh yeah that’s been happening to me’ and I hadn’t put the two together (109_Female_54yrs; CABG; 0-3 months into treatment)

Most participants reported challenges in accessing expert and trusted medication information. Several thought not enough information was given after discharge into the community. Information about the care pathway meant to be followed once in the community was ‘a bit sort of vague […] there is a lack of communication to the individual post-op’ (108_Male_55yrs; CABG; 0–3 months into treatment)

Several participants discussed the need for individuals and families to look for medication information themselves to find answers to their concerns.

Once you are away from the control of the hospital, […] I don’t think patients are necessarily highlighted with (medication side-effects and treatment duration), if they don’t look themselves. […] (You) have to read what you are taking and see in perhaps six months am I taking it for too long etc. (204_Male_61yrs; PCI; 9 to 12 months into treatment)

Apart from that they explained what (the medication) were […] (and) you may bleed a bit more, there was nothing else said about any other side effects but my wife is very nosey and she googles everything so we learnt quite a lot (106_Male_55yrs; PCI; 0 to 3 months into treatment)

Others obtained information from friends and acquaintances.

My wife has a (friend) that’s a dentist and he’s had every kind of heart treatment and has got a defib fixed in so he’s a sort of wealth of knowledge on things. So that’s my first port of call to be quite honest. (108_Male_55yrs; CABG; 0–3 months into the treatment)

Symptom recognition, keeping informed and addressing medication concerns were challenging for participants with complex treatment needs, such as those taking multiple medication for more than one chronic condition.

When you take a cocktail of medication you don’t know which one is doing what so you can’t really answer that (whether nuisance bleeding is caused by DAPT) fully. (110_Male_80yrs; CABG; 0–3 months into treatment)

Others, particularly those coming to the end of treatment, described the need to monitor medication closely, for example being aware of possible interactions between agents or side effects that would potentially need medical attention.

I do read all the literature in tablets now, because I am on 23 tablets a day so I am worried that how do (health professionals) know that one isn’t reacting with another one down the line (205_Male_72yrs; PCI; 9 to 12 months into treatment)

Involvement in care of several health professionals, delivering care crossing primary, community and secondary care settings, was another challenge to information communication. Gaps in communication of information between professionals, ultimately compromised the information communicated to patients.

Once you go back out again (into the community), there is also a question of communication. […] For instance (I have visited) A & E and a couple of times the (local hospital), they have no notes of the heart issues and medication (prescribed in secondary care settings) and (the local hospital) do not write to the patient and occasionally to the GP (204_Male_61yrs; PCI; 9 to 12 months into treatment)

General practitioners (GPs) were the main source of medication information in the community. Many, however, believed GPs did not always have expert knowledge required to advise on complex medication regimens such as DAPT. Participants recounted GPs were not always able to address concerns or felt their GP’s advice contradicted that given by specialists.

There were issues when (medication management was) outsourced to the GP because the GP […] suggested well you could just stay on the Clopidogrel because it might help strokes. […] And it’s sort of an issue of well that’s not what the heart experts are saying. (204_Male_61yrs; PCI; 9 to 12 months into treatment)

Waiting lists for seeing specialists and waiting time between follow-up appointments however acted as barriers to accessing specialist care.

Hospital (appointments) is sort of, every nine months I get a note saying (206) you are on this, you are going to see them and in the meantime if anything happens, you can’t phone them up (206_Male_70yrs; PCI; 9 to 12 months into treatment)

## Discussion

We reported findings from a study exploring the impact of nuisance bleeding on patient DAPT adherence and access to medication information. Almost half of all participants reported experiencing nuisance bleeding, but most thought symptoms to be mild and manageable. Patient and family knowledge and understanding of DAPT, and access to information to address concerns were central to nuisance bleeding responses, regardless of coronary intervention group and treatment duration group. Factors impacting on knowledge and understanding included access to patient-centred medication counselling while in hospital and, access to timely, trusted and appropriate information when in the community. Content of medication counselling delivered in hospital was not always consistent, even when provided in the same care setting. Once in the community, participants found it difficult to find appropriate and trusted information, and often had to take control in monitoring and managing their medication. This was challenging when monitoring complex treatment. Opportunities to access information were hindered by gaps in continuity of care and in the communication of information between secondary and community/primary care settings. Even though overall care fell under the responsibility of GPs, participants had low confidence in medication advice given by GPs.

### Research considerations and implications for clinical practice

Our findings mirror previous studies highlighting the role of patient medication knowledge in encouraging adherence,^11 19^ and the need for education interventions that promote medication literacy, rather than simply impart information.^2 12 14^ Medication literacy is a medication-specific aspect of health literacy discussed in the context of medication adherence, and medication side effects.^20^ It refers to ‘the degree to which individuals can obtain, comprehend, communicate, calculate and process patient-specific information about their medications to make informed medication and health decisions’.^20^ Family members were actively involved in medication management and adherence, and in accessing information, while social networks were important sources of emotional, informational and practical support. Findings reiterate the role of social networks in adherence,^21^ the limitations of focusing on individual health literacy levels,^22^ and the need for a family-centred approach when designing education interventions.^1 2^

Patients need to be informed of postdischarge DAPT risks to increase awareness and inform help-seeking behaviours,^5^ but there are challenges to effectively communicating information when patients are not in frequent contact with the healthcare system in the long term.^12^ So far, patient education interventions have mainly been professional, rather than patient-led, and have focused on short-term, knowledge-specific outcomes. Interventions have not taken into account how adherence is impacted on by patient needs and preferences, and the context in which patients are self-managing their condition for example, patient–doctor relationships and delivery of care.^2 14^

Our study identified contextual factors impacting on medication literacy, relevant when designing medication education interventions. Lack of patient-centred medication counselling meant individual informational needs were not always addressed, for example, women-specific needs. Participants’ psychological and physical state at the time counselling was offered was another factor compromising ability and motivation to engage during the information exchange process. Such findings highlight limitations of patient counselling in hospital settings,^23^ and emphasise the need for appropriately timed, needs-based counselling.^24^ Frequent contact with health professionals throughout patients’ treatment may address medication-specific concerns and information gaps.^20^

Many of our participants preferred to access specialists for medication management, but GPs in primary care settings had overall care responsibility. Participants in many cases had low confidence in the advice given by GPs, who they believed lacked an expert understanding of their treatment regimes. Long-waiting times to see specialists impacted on access to secondary care. Only a small minority of participants had attended cardiac rehabilitation sessions, and these described these sessions as enabling access to expert information and social support. Clinical care pathways allowing for continuity of care across secondary and primary care boundaries are important for enabling patients to access information and meet demands arising from self-managing their care.^25^ A better understanding of patients’ medication management experiences along the secondary and primary care continuum, and with rehabilitation interventions, can inform care delivery and (re)designing of care pathways.^26^

There is a need to better understand patients’ perspectives of the spectrum of pharmacotherapeutic agents they are prescribed, collectively and individually. Polypharmacy hindered symptom recognition and compromised care experiences during interactions with health professionals. These findings reflect others highlighting polypharmacy^27^ and comorbidity^28^ as risk factors for medication non-adherence in patients with heart disease. Participants were more likely to report intentions to make changes to medication regimens other than DAPT, for example, statins, and held more negative attitudes towards such medication. It is not clear why patients hold opposing attitudes towards these agents, or what informs their risk and benefit assessments. Studies have reported disabling symptoms to result in discontinuation and non-adhering to the regimen symptoms are attributed to,^23^ whereas the prospect of taking lifelong medications can also decrease adherence motivation.^24^ Other studies have highlighted a negative portrayal of statins in the UK press^29^ as a factor in patients’ negative attitudes towards statins.^30^

### Strengths and limitations

All research stages were grounded in patients’ experience, thus addressing calls for research facilitating patient and family-centred interventions.^1 2^ Recruitment targeted patients who had undergone three different coronary interventions, and at the early and end stages of DAPT to capture different experiences and perspectives. Focus groups allow for elucidating the range of views and experiences present in a group of participants,^17^ but may suppress or censor individual perspectives or topics that might deviate from those adopted by the group.^17^ Perspectives emerging from this study are also specific to individuals choosing to participate in the study, for example, individuals physically able to travel to and from focus groups and participate in a relatively lengthy group discussion. The physical demands of participating might have prevented more frail patients from attending. Future research should explore individual patient experiences in more depth.

Findings were presented to and endorsed by the ADAPTT study PPI group giving credibility to findings. Only one woman took part in our study, reflecting the under-representation of women in heart disease research.^31^ Participants represented areas of both high and low socioeconomic deprivation, but all were from a white English ethnic background. We highlight the need for more research to explore women’s needs^31^ and patients from ethnic minority backgrounds whose experiences of heart disease^32^ and health literacy^22^ can differ from those of the majority population.

## Conclusions

Positive attitudes towards adherence to DAPT were facilitated by knowledge and understanding of medication, and ability to deal with symptoms and minimise impact on quality of life. Opportunities to access medication counselling and address concerns after discharge from hospital were hindered by lack of a personalised approach to medication counselling in hospital settings, discontinuities in care provision across primary and secondary care settings, and a lack of trust in GPs’ expertise. Interventions should adopt a participatory, coproduction approach at all stages of intervention design so that they address the needs, preferences and priorities of patients and their families.^14^ Design of interventions should also be informed by a family-centred approach to medication literacy that acknowledges the role of families in self-management and adherence.^1^
