# Supplementary material for: Patient perspectives of nuisance bleeding and adherence to dual antiplatelet therapy: a qualitative study
Source: Open Heart. 2020 Oct 15;7(2):e001405. doi: 10.1136/openhrt-2020-001405 (PMC7566423; doi:10.1136/openhrt-2020-001405)
Supplement: Supplementary data [file openhrt-2020-001405supp001.pdf]

Patient focus groups
Topic guide

- **Thank you for coming along**
- **Introduce facilitator and co-facilitator.**
- **Set ground rules e.g. respect each other's opinions**
- **Invite each participant to introduce themselves, giving first name only.**

The facilitator will encourage discussions around **participants' healthcare-seeking behaviour in relation to bleeding while on DAPT**. The questions and prompts below will be used flexibly to stimulate responses and discussion.

1. **Information about DAPT**
   a. **Participants' level of knowledge of DAPT**
   - Were you told about what your medication does while you were in hospital?
   - *Were you told about the side effects? Did you understand what you were told?*
   - Were you clear about how long to take the medication for?
   - Did you read the information leaflets? How helpful did you find them?
   - Did you access any other information (internet, healthcare professionals, friends and family)?
   - *Would you have liked more information? In what format?*
2. **Issues related to adherence**
   - Do you try and take your medication regularly as prescribed?
   - When might you miss a dose?
   - What do you do if you miss a dose?
   - What are the barriers/facilitators to taking your medication regularly as prescribed?
3. **Issues related to bleeding**

As some of you have discussed, some people develop some bruising and bleeding as a result of taking the medication.

- Has this happened to you? What sort of bleeding did you experience?
- Did/ would this cause you concern?
- Did/ would you contact your GP (or another healthcare professional) about this?
- At what point would bleeding become a problem for you?

CLOSING DISCUSSION

Take it in turns to raise the most significant ways in which this medication has impacted/you expect it to impact on your quality of life, whether in positive, or negative ways

IRAS No: 203302; ADAPTT Qualitative Study; Patient focus group topic guide; June 2017
